# Insight into a pure spinel Co_3_O_4_ and boron, nitrogen, sulphur (BNS) tri-doped Co_3_O_4_-rGO nanocomposite for the electrocatalytic oxygen reduction reaction

**DOI:** 10.1039/d3ra04600a

**Published:** 2023-10-03

**Authors:** Afia Kanwal Bhatti, Naila Jabeen, Amna Bashir, Latif U. Khan, Syeda Wishal Bokhari, Zareen Akhter

**Affiliations:** a Department of Chemistry, Quaid-i-Azam University Islamabad 45320 Pakistan Zareenakhter@yahoo.com Zareen_a@qau.edu.pk; b Nanoscience's and Technology Division, National Center for Physics, Quaid-i-Azam University Campus Shahdra Valley Road, P.O. Box 2141 Islamabad 44000 Pakistan naila.chem@gmail.com naila.jabeen@ncp.edu.pk; c Department of Chemistry, Fatima Jinnah Women University The Mall Rawalpindi Pakistan; d Synchrotron-Light for Experimental Science and Applications in the Middle East (SESAME) P.O. Box 7 Allan 19252 Jordan; e Ningbo Institute of Materials Technology and Engineering, Chinese Academy of Sciences Ningbo 315201 Zhejiang People's Republic of China

## Abstract

The intricate problems concerning energy require innovative solutions. Herein, we propose a smart composite nano system that can be used in a sustainable and dichotomous manner to resolve energy crises. The current study describes a new way to synthesize a pure spinel cobalt oxide (Co_3_O_4_) and boron (B), nitrogen (N), and sulfur (S) tri-doped Co_3_O_4_-reduced graphite oxide (rGO) nanocomposite (CBNS). A hydrothermal method has been used for the synthesis of these nanomaterials. The synthesized nanocomposite was characterized by UV-visible spectroscopy, X-ray diffraction (XRD), Raman spectroscopy, scanning electron microscopy (SEM), X-ray absorption spectroscopy (XAS), and transmission electron microscopy (TEM). The XRD results showed the formation of Co_3_O_4_ and B, N, S doped nanocomposite with high purity and crystallinity. XAS analysis elucidates the formation of spinel Co_3_O_4_ with tetrahedral and octahedral arrangement of cobalt ions. The peaks at 2.50 Å and 3.07 Å are due to the Co–Co bonding. The electrocatalytic oxygen reduction (ORR) was successfully implemented using these nanocomposites. The electrochemical study exhibits the better activity of the B, N, and S tri-doped Co_3_O_4_-rGO nanocomposite due to the mutual effect of B, N and S. The synthesized catalyst has maximum current density of 9.97 mA cm^−2^ with onset potential (*E*_onset_) of 0.98 V in alkaline medium.

## Abstract 2

Herein, we propose a smart composite nano system that can be used in a sustainable and dichotomous manner to resolve energy crises.

## Graphical abstract

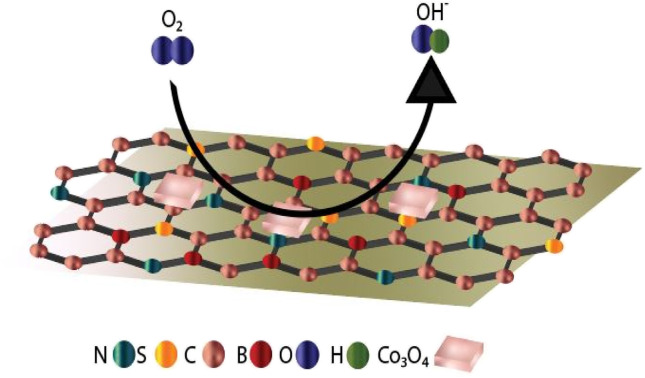

## 1. Introduction

The issue of renewable energy is captivating due to intensifying power demand, rising oil costs and environmental complications.^1^ To attain a safe and sustainable future it is necessary to introduce some innovations into the existing renewable energy conversion and storage systems.^2^ Out of the innumerable renewable energy devices, fuel cells are the supreme way of energy exhibiting high productivity, light operation process, zero emanation, and most importantly unlimited continual source of reactants.^3,4^ An electrochemical device known as a fuel cell transforms chemical energy into electrical energy. Typically, oxygen is reduced at the cathode, and hydrogen is oxidized at the anode in a fuel cell.^5,6^ The most important reaction of fuel cells is the reduction of oxygen (ORR) which is a big challenge in the domain of electrochemistry.^7^ Due to the sluggish kinetics of this reaction, it demands a catalyst.^8,9^ So far, platinum (Pt) and derivatives of Pt have been used due to their high exchange current density, better stability, and higher catalytic activity.^10–12^ But their high cost and finite resources have limited their use practically. There have been numerous attempts to substitute these with noble metal catalysts, but researchers continue to face significant challenges with the activity and stability of the catalyst.^13^

Out of all of the non-noble metal-based catalysts, spinel cobalt oxide has attracted researchers due to its easy availability, cost-effectiveness, low overpotential, best performance, and durability during electrochemical reactions. Cobalt oxide is also an active catalyst for methane combustion, carbon monoxide oxidation at low temperatures, anode material in lithium-ion batteries, and a gas sensor.^14,15^ It is sometimes agglomerated and has poor conductivity due to it reduce surface area and massive particle size. This will affect the activity of the catalyst material. Different strategies are adopted to enhance the efficiency of the catalyst, one of which is to make nanostructures or composites of the catalyst and second is to use conductive material like graphite, graphene, graphene oxide, carbon nanotubes, mesoporous carbon *etc.*^16,17^

Graphene is a new class of material constituting carbon atoms that are covalently bonded to three other carbon atoms. It is an attractive substance because of its excellent optical, thermal, mechanical and electrical properties.^18^ Other than these, materials of graphene have large surface area, maximum stability, high chemical tolerance, structural flexibility and reassembly properties which are advantageous for electrochemical devices.^19,20^ Due to their increased surface area, organized balance structure and native metallic properties these carbonaceous materials would aid the increment of electronic conductivity and dispersal of metal compounds. The activity of graphene oxide is further enhanced by doping with different non-metals like oxygen (O), boron (B), nitrogen (N), phosphorus (P), and Sulphur (S).^21^ These heteroatoms provide anchoring sites by facilitating the coordination of metal ions with carbon atoms creating M–O–C, M–N–C bonds.^22^ For example, Meng *et al.* incorporate N and S in graphene with cobalt oxide as an efficient catalyst for ORR and OER.^44^ Wei *et al.* modify graphene tubes with N and cobalt for ORR.^45^ S. Fajardo *et al.* study the effect of dual (N & S) doped graphene with spinel cobalt oxide for ORR.^46^ Dai's group reported high electrocatalytic activity of Co_3_O_4_ grown on rGO for ORR and oxygen evolution reaction (OER). This high activity was due to the synergistic effect between cobalt oxide and graphene oxide. They also studied the result of nitrogen doping in graphene oxide and found that N doping will enhance the activity of Co_3_O_4_/N-rGO. This high activity was due to bond formation between Co–O–C and Co–N–C.^23^ Tong *et al.* prepared a hybrid material consisting of CoO_*x*_ and B, N doped graphene. This composite material is beneficial for ORR and OER due to oxygen vacancies and Co–N–C bond.^24^ Lu *et al.* synthesized a composite of cobalt with B, N, S doped graphene and investigated this catalyst for ORR.^22^ Anita Swami and her group designed a unique catalyst of cobalt monoxide with B, N, S doped rGO which served as dual purpose catalyst for ORR and OER.^25^ Liu *et al.* studied spinel NiCoO nanosheets as a electrocatalyst for ORR.^26^ Pu studied different faces of spinel cobalt oxide for ORR.^27^ Liang *et al.* observed nanocrystals of Co_3_O_4_ supported on graphene as a promising catalyst for ORR.^28^ Buchner *et al.* reported mixed metal oxides (Co_3_O_4_ with Ni and Mn) with low overpotential and higher current density than Co_3_O_4_ alone.^29^

Herein, we successfully synthesized a novel, low-cost and stable B, N, and S doped rGO nanocomposite with Co_3_O_4_ for ORR. The morphology of synthesized nanocomposite was carried out using (SEM) and (TEM). To confirm the oxidation of graphite and to analyze the crystal structure of electrocatalyst X-ray diffraction (XRD) technique has been used. Raman spectroscopy differentiates the graphite from graphite oxide. Metal's local environment and oxidation state were verified using X-ray absorption spectroscopy (XAS). To our best knowledge, this is the new approach about the synthesis of tri-doped rGO nanocomposite with spinel Co_3_O_4_ and its detailed analysis has been done for the first time in this study.

## 2. Experimental

### 2.1. Synthesis of Co_3_O_4_ and undoped GO

Co_3_O_4_ was synthesized using the method already reported in literature with slight modifications. Briefly, 0.3 molar solution of cobalt was prepared by adding 3.6 mg cobalt acetate hexahydrate Co(CH_3_COOH)·6H_2_O in 50 mL ethanol. The above solution was stirred at 25 °C for 1/2 hrs. The solution was then transferred to two-neck flask. The temperature of solution was raised to 50 °C with continuous stirring for 30 min. 3.6 mL of oxalic acid is added to the above solution. At 50 °C, the mixture was refluxed for two hours. The resultant pink precipitate was filtered and washed with ethanol twice. The precipitates were first dried at 80 °C before being calcined for 2 hours at 450 °C.^30^

Original Hummers' method was used to prepare graphene oxide. Briefly, 0.50 g of sodium nitrate (NaNO_3_) and 1 g of graphite powder was dissolved in 23 mL of sulfuric acid (H_2_SO_4_) and stirred in an ice bath. With continuous stirring, 3.00 g of potassium permanganate (KMnO_4_) was added to the above suspension carefully so that temperature of the suspension should not exceed from 20 °C. After removing ice bath, the temperature of the above suspension was maintained at 35 °C for 1/2 hrs. After 30 min, there was a slow addition of 46 mL of water to the brownish-grey paste. Due to this addition, the temperature rises to 98 °C and there was a strong effervescence. Then the diluted suspension was stirred at 98 °C for 15 min. After adding 140 mL of warm water, the suspension was further diluted before being treated with 3% hydrogen peroxide (H_2_O_2_). In the end, the suspension turned bright yellow with the treatment of peroxide. The suspension was filtered and washed with warm water three times. The resulting graphite oxide was cured for four hours at 40 °C.^31,32^

### 2.2. Synthesis of B, N, and S doped GO

B, N, and S doped graphite oxide was created using a hydrothermal technique. In this method 1 g boric acid and 0.50 g l-cysteine was mixed in 2.50 mL DI water and heated at 70 °C for 2 h. After heating a yellow colored solution was obtained, and then this solution was heated in an autoclave at 200 °C for 3 h. At last, the obtained solution was diluted using DI water to make 100 mL solution. The graphite oxide was dispersed in solution of B, N, and S by ultra-sonication for 30 min. The sample was filtered after sonication of 30 min and then dried at 60 °C. B, N, and S was homogeneously distributed on GO and denoted as BNS/GO.^25^

### 2.3. Synthesis of Co_3_O_4_/rGO nano-composite

**Fig. 1:**
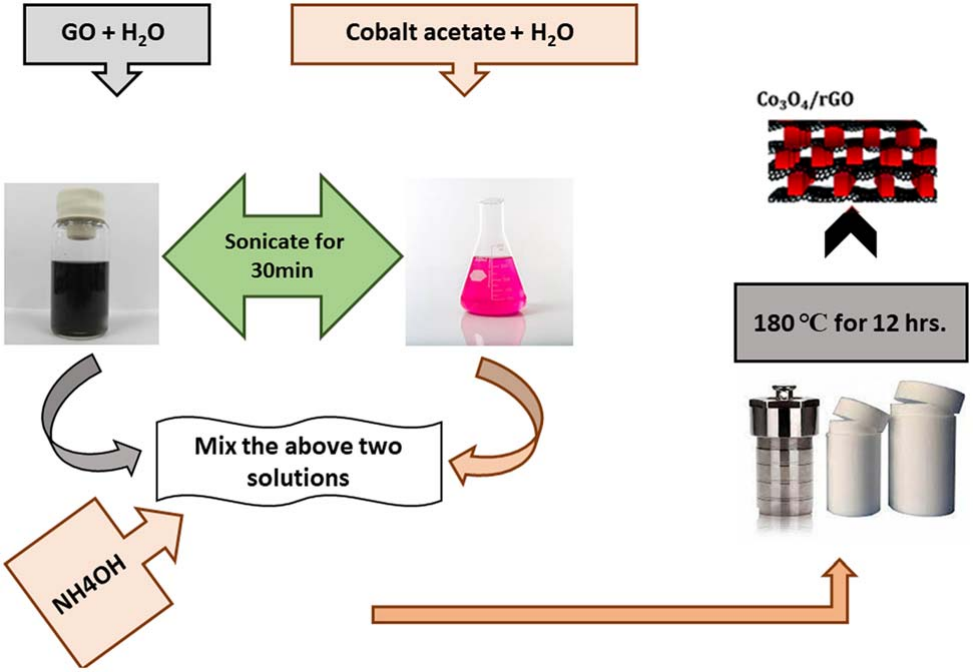
Schematics representation of synthesis of Co_3_O_4_/rGO nano-composite.

To prepare Co_3_O_4_/rGO composite (RGC), 0.465 g of cobalt acetate tetrahydrate was solvate in 10.0 mL distilled water Fig. 1. GO (0.15 g) was dissolved in 125 mL of water through sonication for 2 h to attain uniform distribution of GO. Above two solutions were mixed and added with 5.00 mL of NH_4_OH to precipitate cobalt ions and to reduce GO. Then obtained mixture was put into an autoclave for hydrothermal reaction at 180 °C for 12 h. Then after centrifugation the resultant material was collected and then washed with DI water to eliminate any contamination particles and purify by washing with ethanol three times.^33^

Spinel Co_3_O_4_ decorated on B, N, and S doped GO sheets was also done by hydrothermal method. Briefly, 0.23 g Co(CH_3_COOH)_2_·4H_2_O was dissolved in 5.00 mL DI water to make solution A, and 0.075 g B, N and S doped GO was added to 63.0 mL DI water to make solution B. The solution A was mixed with solution B followed by the addition of 5.00 mL of ammonia solution. Then obtained mixture was put into an autoclave for hydrothermal reaction at 180 °C for 12 h. Then after centrifugation the resultant material was collected and then washed with DI water to eliminate any contamination particles and purify by washing with ethanol three times.

Using a Bruker APEXII D8 Venture diffractometer equipped with photon 100 detectors and Cu k_α_ radiation, single crystal XRD analysis was performed. The samples were scanned between 2*θ* = 5–80 °C diffraction angle. XRD was employed to confirm the oxidation of graphite and to analyze the crystal structure of electrocatalyst. The SEM images were taken from JEOL JSM-7600F field emission scanning electron microscope. The TEM images were obtained from JEOL TEM 2010 and 2100F. With the 532 nm laser excitation, on a Renishaw Raman analysis was performed using a TM reflex micro spectrometer. The middle East XAFS/XRF beamline synchrotron light for experimental science and applications evaluated the X-ray absorption fine structure XAFS spectra (SESAME). Using a PerkinElmer Lambda 35 UV-visible spectrophotometer, the optical absorbance data and band gap energies were determined.

The XAFS measurement was carried out on the XAFS/XRF beamline,^42^ at the middle eastern synchrotron light for experimental science and applications, which was operating in decay mode at 2.5 GeV and with a maximum electron current of 300 mA at room temperature, in transmission mode XAFS spectra were collected, in the spectral ranges of Co K-edge (7709 eV) for the Co_3_O_4_, CBNS and RGC samples with a double-crystal Si (111) monochromator. The energy of the monochromator was calibrated with standard Co foil. Three ionization chambers (for beam intensity measurements) were filled with optimal Ar/N_2_ gas mixtures at a total pressure of 1.0 bar, and the signal measured at the ion chambers was amplified by Stanford picoammeters, digitalized by a voltage to frequency converter, and then read by the counters at the PXI-NI to obtain the XAFS data of the samples and standard Co foil. The samples were created by pressing (less than 2 tons) a homogeneous mixture of the prescribed amount of powdered polyvinylpyrrolidone PVP and finely ground material (less than 5 μm) into pellet form(13 mm diameter). The amount of material in each pellet was calculated using XAFS mass software to give the absorption *μ*_t_ ∼ 1.5, just above the rare earth (Eu, Gd, Sm) L_3_-absorption edges.

### 2.4. Electrochemical measurements for ORR activity

The electrochemical application of all synthesized electrocatalysts was examined using a CHI 660D electrochemical workstation using three electrode system. The experiment was performed using Ag/AgCl as reference electrode and platinum wire as counter electrode. All measurements were carried out in 0.1 M KOH solution at 25 °C. Before performing any experiment, the working electrodes were polished using alumina powder. Then electrodes were rinsed with distilled water and acetone and then dried.

For working electrode ink was prepared by adding 3.00 mg of catalyst in 1.00 mL ethanol containing 10.00 μL nafion, followed by sonication for 2 h at room temperature. This ink was coated on working glassy carbon electrode by using micropipette. The electrode was dried overnight before carrying any measurements. The electrode potential values were changed with respect to reversible hydrogen electrode (RHE) by using formula given below.

1 *E*_RHE_ = *E*_Ag/AgCl_ + 0.059 pH + *E*^0^_Ag/AgCl_

where *E*_RHE_ is the converted potential *vs.* RHE, *E*_Ag/AgCl_ is the experimental potential measured against Ag/AgCl reference electrode, and *E*^0^_Ag/AgCl_ is the standard potential of Ag/AgCl at 25 °C (0.1976 V). LSV (ORR) was performed at scan rate of 50 mV s^−1^.

## 3. Results and discussion

### 3.1. Characterization of spinel Co_3_O_4_ and GO

**Fig. 2:**
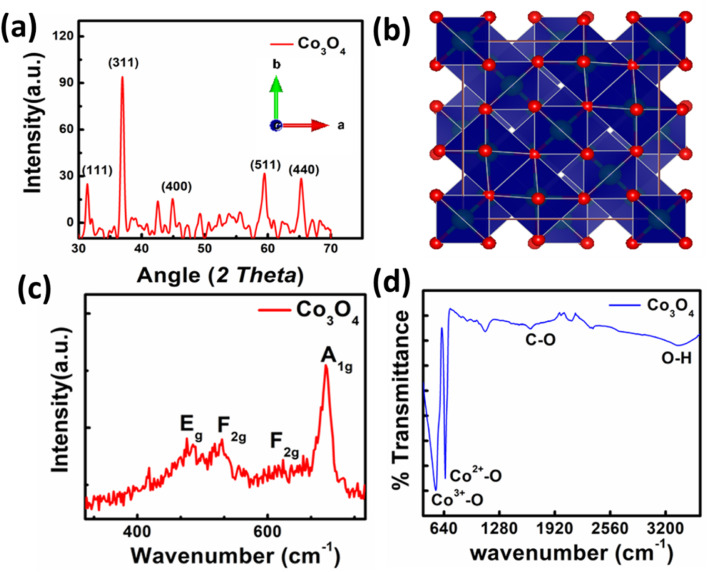
(a) XRD pattern of nanoparticles, (b) crystal structure of Co_3_O_4_, (c) Raman spectra of Co_3_O_4_ nanoparticles, (d) FTIR spectra of Co_3_O_4_.

The synthesized material was characterized using XRD. The XRD pattern of synthesized spinel Co_3_O_4_ nanoparticles is shown in Fig. 2a. The peaks appeared at 2*θ* values of 31.31°, 36.81°, 44.93°, 59.72° and 65.27° are corresponding to the (220), (311), (400), (422) and (440) reflections. The peaks appearance provides evidence that spinel Co_3_O_4_ nanoparticles has successfully formed. The experimental data is in good agreement with the reference database card of JCPDS 09-0418. Furthermore, absence of peaks related to CoO, and Co(OH)_2_ confirms the phase purity of synthesized nanoparticles. The crystallite size of Co_3_O_4_ is also calculated using Debye–Scherrer equation.^34^ The calculated average crystallite size is 19.00 nm. The crystal structure of spinel cobalt oxide is shown in Fig. 2b.

The Raman spectrum of synthesized Co_3_O_4_ is presented in Fig. 2c. In the Raman spectrum of spinel cobalt oxide four bands appeared *i.e.*, at 482 cm^−1^, 525 cm^−1^, 615 cm^−1^, and 686 cm^−1^ respectively. The first band is corresponding to the E_g_ mode, while the later two are assigned to the F_2g_ modes. The band at 686 cm^−1^ is ascribed to the A_1g_ vibrational mode. These bands confirm the formation of Co_3_O_4_ nanoparticles. The FTIR spectrum of Co_3_O_4_ is shown in Fig. 2d. The spectrum reveals that broadband at 3379 cm^−1^ is attributed to symmetric and asymmetric vibration of O–H of water. The band at 1642 cm^−1^ is due to the asymmetric vibration of C–O. The bands at 545 cm^−1^ and 651 cm^−1^ are attributed to Co^3+^–O and Co^2+^–O stretching respectively.

**Fig. 3:**
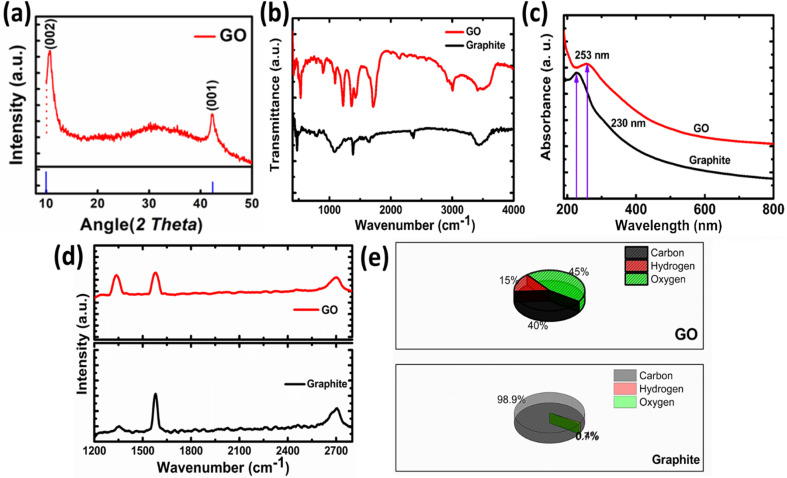
(a) XRD pattern of GO (b) FTIR, (c) UV-vis spectra, (d) Raman, (e) (CHNS) analysis.

The XRD pattern of GO is shown in Fig. 3a. The peak appeared at 11.96° is the characteristics peak for GO corresponding to the interplanar spacing of 0.74 nm. In case of graphite this peak appeared at 24.6° corresponding to the (002) reflection with interplanar spacing of 0.335 nm.^32^ The increase in the interlayer spacing confirms the presence of oxygen containing moieties.^31^ These oxygen containing moieties cause increase in interplanar distance and reduction in 2*θ* value. Hence, preliminary confirmation for the synthesis of graphite oxide was obtained by XRD. Only one peak in GO indicates that highly oxidized form has been obtained to about 0.74 nm also confirms the expansion and oxidation of graphene.

The FTIR spectrum of graphite oxide is shown in Fig. 3b. The peak appeared at 1408 cm^−1^ is due to the symmetrical vibration of OH group. While the peak that appeared at 1630 cm^−1^ is ascribed to the C–O–C vibration of epoxide groups. A broad peak appeared between 2400 cm^−1^ to 3600 cm^−1^ corresponding to the hydrogen-bonded OH vibration in COOH groups. The strong peak that appeared at 1730 cm^−1^ in GO confirms the formation of GO, as this peak is absent in graphite. This peak is due to the carbonyl stretching vibration in the COOH group.^35,39^ The UV-visible spectroscopy is used to confirm the formation of GO further as shown in Fig. 3c. The shifting to UV peak from 230 nm (graphite) to 253 nm in the case of GO confirms the formation of GO from graphite. This peak corresponds to the π electron conjugation in the molecule. This value matches the reported value for GO.^36^

The Raman spectrum of Graphite and GO is shown in Fig. 3d. The GO is characterized by the appearance of two bands in Raman spectra. The band appeared at 1600 cm^−1^ is known as G band corresponding to the C–C vibration of sp^2^ hybridized carbon, while the band at 1350 cm^−1^ is called as D band indicates the vibrations of carbon atoms at edges of the free separated graphite sheets.^37^ The G band is the indication of ordered graphite oxide sheets while D band indicates the disordered or scattered graphite sheets. In case of GO the intensity difference between D and G band is prominent as compared to the pristine graphite. The D/G band ratio in graphite is 0.22, while in case of GO it is 0.95 which is the good indication for oxidation and exfoliation of graphite.^31^ The samples were further analyzed by measuring hydrogen, oxygen and carbon contents as shown in Fig. 3e. The graphite sample contains 98.9% carbon, 0.74% oxygen, and 0.74% hydrogen. A small percentage of hydrogen and oxygen come from adsorbed water or oxygen-containing functional groups. In the case of GO oxygen and hydrogen contents increased up to 45% and 40%.

**Fig. 4:**
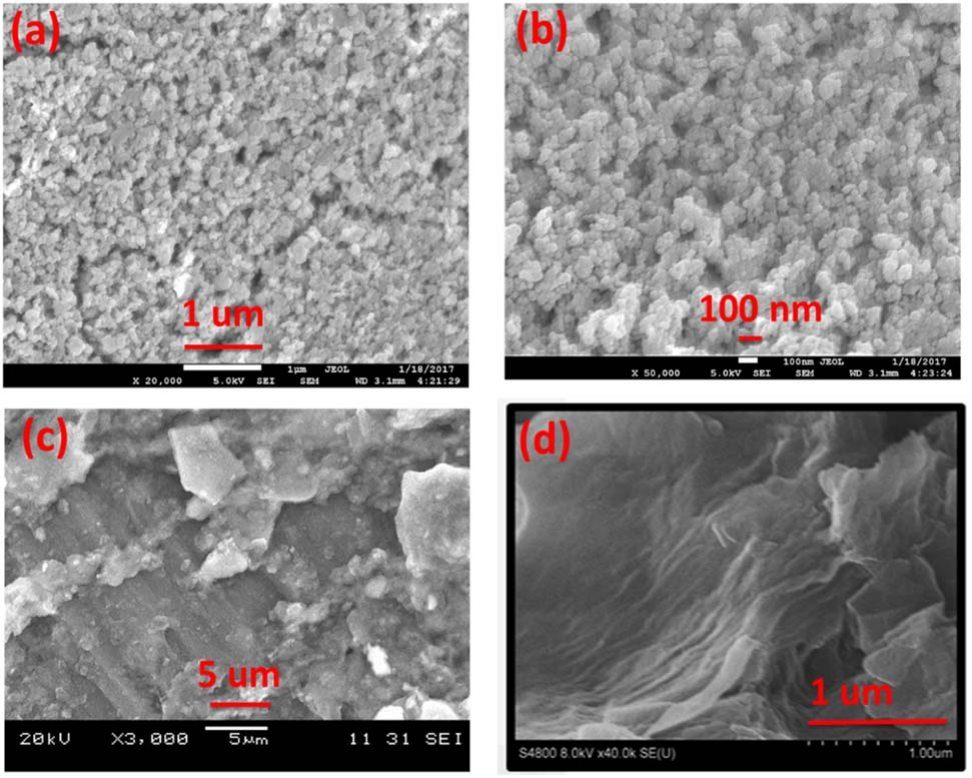
SEM images different magnification of (a and b) Co_3_O_4_, (c and d) graphite and GO.

The morphology of synthesized Co_3_O_4_ nanoparticles and GO was evaluated by FE-SEM as shown in Fig. 4. The Fig. 4a and b showed that the synthesized Co_3_O_4_ are almost spherical in shape and are slightly agglomerated. The average particle sizes are appeared to be less than 100 nm. In case of synthesized GO Fig. 4d the sheets exhibit sheet/leaf like morphology. Furthermore, it is clear from images that the GO sheets are highly folded which may be due to the interaction of various oxygen functional groups between the layers of GO.^35^ This folded pattern is not seen in the SEM image of graphite Fig. 4c.

### 3.2. Characterization of Co_3_O_4_/rGO, BNS-rGO and Co_3_O_4_/BNS-rGO nanocomposite

**Fig. 5:**
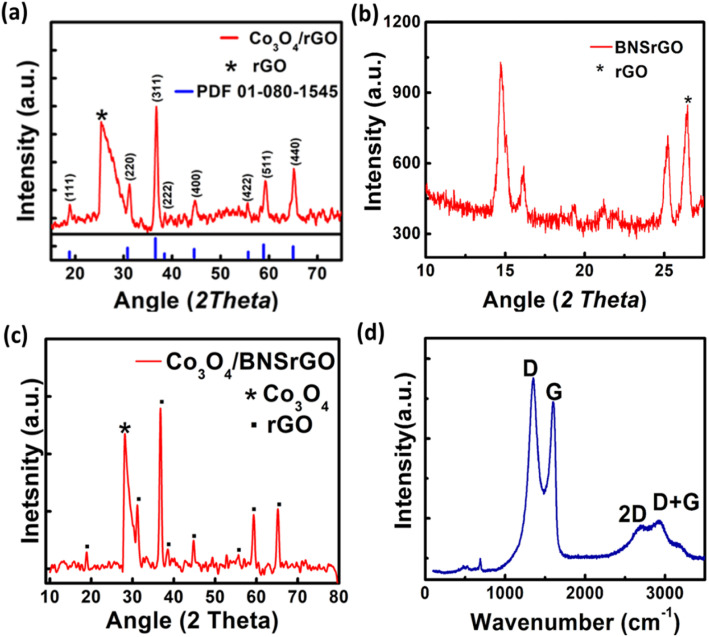
XRD pattern of (a) spinel Co_3_O_4_/rGO, (b) BNS rGO, (c) spinel Co_3_O_4_/BNS-rGO, (d) Raman spectra of Co_3_O_4_/BNS-rGO.

The XRD pattern of Co_3_O_4_/rGO, BNS-rGO and Co_3_O_4_/BNS-rGO is shown in Fig. 5a–c. The peak for GO is shifted from 10.78° to 27.05° in all three materials which confirms the conversion of GO to rGO.^38^ The peaks at 2*θ* values of 19.16°, 31.31°, 36.81°, 38.52°, 44.93°, 55.74°, 59.72°, and 65.27° confirms the pure and crystalline Co_3_O_4_ phase Fig. 5a–c ref. 34 (JCPDS 09-0418). The diffraction peaks confirm the absence of any other phase and impurities. The calculated crystallite size of Co_3_O_4_ is 14.0 nm. In the Raman spectrum of a composite of Co_3_O_4_/BNS rGO, Fig. 5d D band remains at the same position while G band shifts at 1610 cm^−1^. This displacement of the band is caused by rGO ability to restore a carbon network with defects through self-healing. The Raman spectrum of the composite show four peaks at approximately 482, 525, 615, and 686 cm^−1^ which attributes to Eg, F_2_g, F_2_g, and A_1_g modes of Co_3_O_4_ nanoparticles.^35,39^

**Fig. 6:**
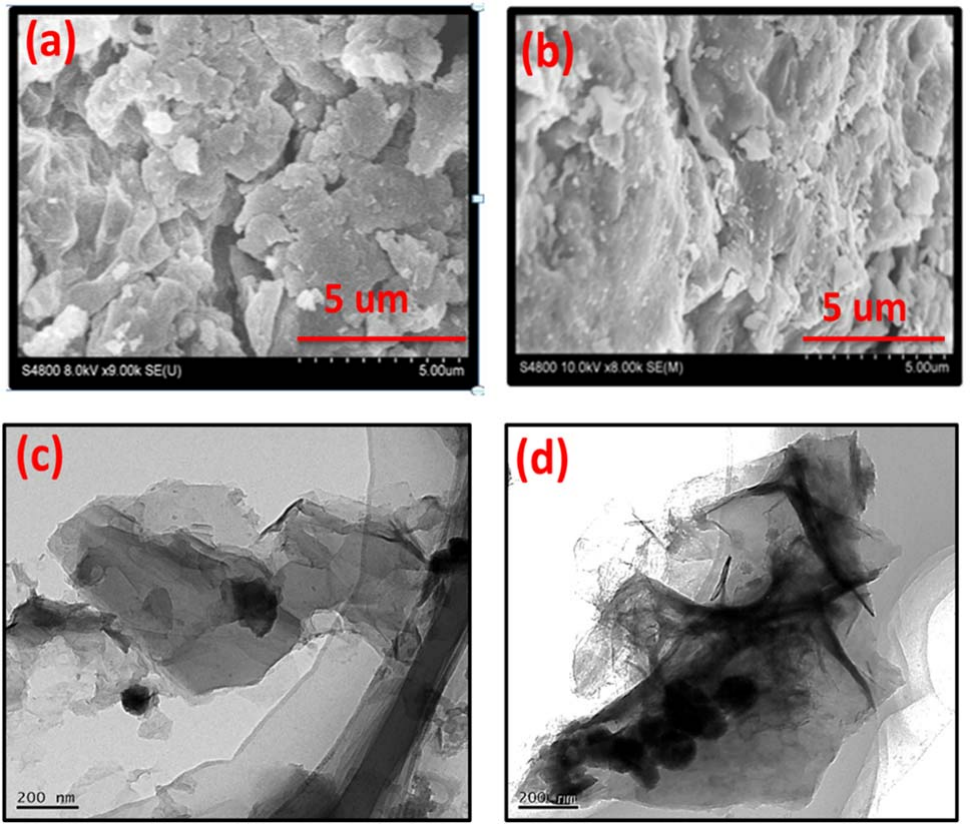
SEM images of (a) BNS rGO, (b) Co_3_O_4_/BNS rGO, (c and d) TEM images of Co_3_O_4_/BNS rGO.

The SEM image of B, N, and S doped GO is shown in Fig. 6a. It is clear from images that the doping with B, N, and S changes the surface morphology of GO. The sheets of GO are more crumbled after doping with heteroatoms. However, in case of Co_3_O_4_/BNS-rGO Fig. 6b the presence of nanoparticles of Co_3_O_4_ on uniform sheets of graphite oxide is observed. These round shaped nanoparticles are homogeneously anchored on silky sheets of graphite oxide with an approximate average diameter of 150 nm. This may be due to covalent coordination of metal with positive charge (Co^2+^, Co^3+^) and negatively charged oxygen containing functional groups of GO. The TEM images confirm the findings of SEM Fig. 6c and d. TEM image of Co_3_O_4_/BNS-rGO reveals that cobalt oxide nanoparticles have a circular shape, and these are arranged in a cluster on sheets of GO. TEM confirms that GO sheets have silky nature and these are separated from each other.

**Fig. 7:**
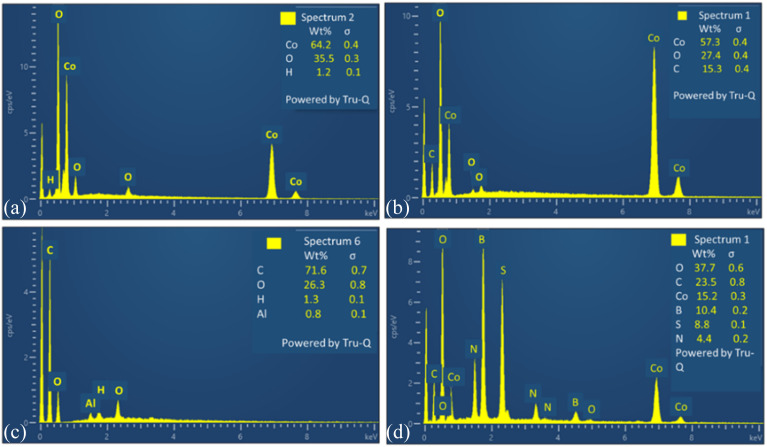
EDX images of (a) Co_3_O_4_, (b) Co_3_O_4_/rGO, (c) GO, (d) Co_3_O_4_/BNS rGO.

To analyze the elemental composition of synthesized materials EDX have been carried out. This confirms the percentage composition of all elements in composite of spinel cobalt oxide with tri-doped rGO. EDX of all electrocatalysts are depicted in Fig. 7.

## 4. Probing local atomic structure of Co_3_O_4_, CBNS and RGA nanostructures by XAFS

**Fig. 8:**
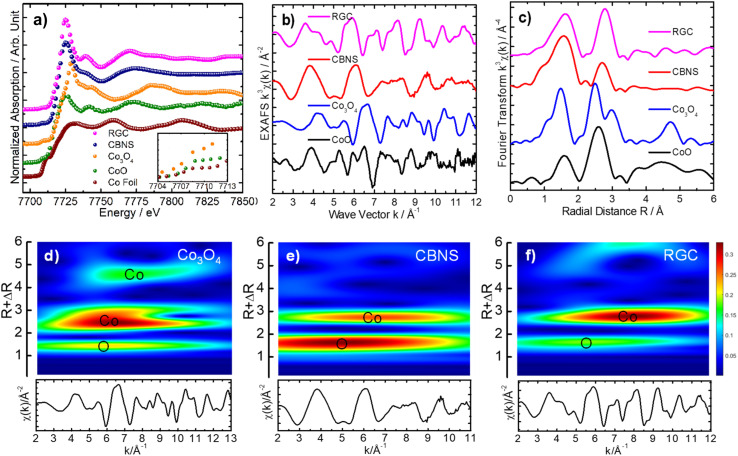
Normalized XANES spectra (a), experimental k^3^-weighted EXAFS signals (b) their respective Fourier transforms (c) and two-dimensional CCWT images (d–f) yielded from the respective experimental k^3^-weighted EXAFS signals, manifesting the localization of O and Co back scatterers as colored maps for the Co_3_O_4_, CBNS and RGA nanomaterials.

The experimental Co K-edge (7709) XANES spectrum of the Co_3_O_4_ catalyst manifested well the mean Co oxidation state between +2 and +3, as expected for the pure Co_3_O_4_ phase,^40^ and showed the weak pre-edge peak A at 7708.8 eV, weaker shoulder at about 7722 eV (B) and a rising edge peak C. The cubic lattice of the Co_3_O_4_ was revealed by the development of a very weak pre-edge peak associated with the 1s-3d quadrupole transition for the Co octahedral site.^41^ Whereas, significant change in the near edge features and shift toward lower energy for the Co_3_O_4_/BNS rGO and Co_3_O_4_/rGO nanostructures were observed, appearing near to the Co K-edge XANES of reference CoO. This result exhibited the presence of considerable amount of Co in +2 oxidation state. In order to explore the difference in local atomic composition of these three nanocatalysts, their EXAFS data were examined. The experimental k^3^-weighted EXAFS oscillations of the Co_3_O_4_ are quite different from the Co_3_O_4_/BNS rGO and Co_3_O_4_/rGO nanocatalysts, demonstrating different local structures at the Co site. The EXAFS oscillations of the Co_3_O_4_/BNS rGO and Co_3_O_4_/rGO showed slight similarity with that of the reference CoO, suggesting a local structure slightly close to the CoO cubic lattice. The Fourier transform of χ(*k*) for the Co_3_O_4_ displayed two dominant peaks, the first peak corresponded to the O backscattered in the first coordination shell and the second peak represents the Co backscattered in the second coordination shell, demonstrating the typical pure cubic phase of Co_3_O_4_ lattice.^40^ However, the comparatively broadness of the first peak (∼1.5 Å), corresponded to the oxygen (O) backscattered (first coordination shell) for the Co_3_O_4_/BNS rGO and Co_3_O_4_/rGO manifested the contribution of O interaction to Co from other lattice also, presumably graphene oxide (GO). The other important structural effect is observed in the second shell within Co–O–Co atomic lattice. Whereas, the successive change in shape of the second peak (∼2.8 Å) for the Co_3_O_4_/BNS rGO and Co_3_O_4_/rGO, and considerable decrease in the amplitude for the Co_3_O_4_/BNS rGO demonstrated clear change for Co site in second shell for these two nanocatalysts, when compared to the Co_3_O_4_. The similar result was also observed in the two-dimensional CCWT images of the Co_3_O_4_/BNS rGO and Co_3_O_4_/rGO, which were qualitatively analyzed through continuous Cauchy wavelet transform (CCWT), using MATLAB 2019b.^43^ The two-dimensional CCWT images of these two nanostructures showed the difference in the shape of the colormaps, corresponding to O backscattered in first coordination shell and Co backscattered in second coordination shell, when compared to those of Co_3_O_4_. Nevertheless, the overall X-ray absorption fine structure spectroscopy (XAFS) data analyses results suggested pure cubic Co_3_O_4_ phase for the Co_3_O_4_, and thus, change in the local atomic structure of the Co site for Co_3_O_4_/BNS rGO and Co_3_O_4_/rGO nanocatalysts, when compared to the Co_3_O_4_ cubic lattice Fig. 8.

## 5. Electrocatalytic activity of synthesized nanocomposite

**Fig. 9:**
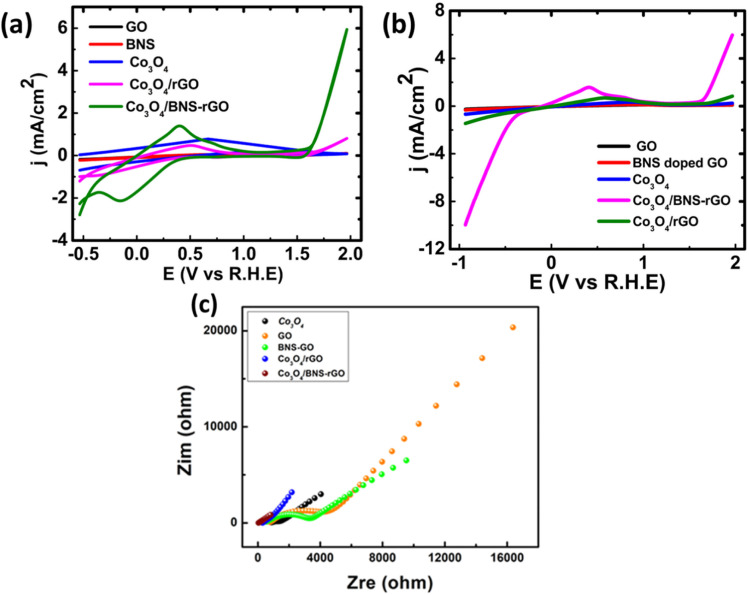
(a) Comparative CV curves of samples in O_2_ saturated 0.1 M KOH at a scan rate of 50 mV s^−1^ (b) comparative LSV curves of different samples in O_2_ saturated 0.1 M KOH at a scan rate of 50 mV s^−1^ (c) comparative Nyquist plots of Co_3_O_4_/BNS-rGO fitted by CPE model (inset) circuit diagram for CPE model.

The catalytic activity of synthesized nanocomposites is tested for ORR. The electrochemical studies for all samples were carried out by using cyclic voltammetry (CV), linear sweep voltammetry (LSV) and electrochemical impedance spectroscopy (EIS). The Fig. 9a shows the cyclic voltammograms of Co_3_O_4_ and Co_3_O_4_/BNS-rGO recorded at scan rate of 50 mV s^−1^ using three electrode systems in 0.1 M KOH solution. Here Pt is used as counter electrode and Ag/AgCl electrode is used as reference electrode, respectively. The presence of reduction peak in CV confirms the electrocatalytic activity of Co_3_O_4_/BNS-rGO. The electrochemical activity of Co_3_O_4_/BNS-rGO electrocatalyst is further studied using linear sweep voltammograms (LSV) in 0.1 M KOH solution at a scan rate of 50 mV s^−1^. The results are shown in Fig. 9b. From these results, it is clear that the Co_3_O_4_/BNS-rGO electrocatalyst have better peak potential (0.99 V) comparable to benchmark Pt/C (0.99 V). It also exhibits a higher current density of −9.97 mA cm^−2^ with an improved peak potential of 0.99 V than Co_3_O_4_/rGO and Co_3_O_4_ without any support and GO alone has current densities of −1.5 mA cm^−2^, −0.67 mA cm^−2^, and −0.21 mA cm^−2^. The improved current density is due to electron-rich dopants (N & S) which provide electrons to metal that will be useful for oxygen reduction. During this electron donation, a metal that exists in two oxidation states (Co^2+^, Co^3+^) in spinel Co_3_O_4_ will be converted from +3 to +2 states.

Electrochemical impedance spectroscopy (EIS) further confirms the best activity of Co_3_O_4_/BNS doped rGO. The Fig. 9c depicts the EIS plot (Nyquist plot) of Co_3_O_4_ and Co_3_O_4_/BNS-rGO. The data plot is recorded between real and imaginary frequency on *X* and *Y* axis respectively and frequency recorded in the frequency range of 1 Hz–100 kHz. The EIS plot gives the charge transfer resistance (*R*_CT_) which provides knowledge about conductivity of the material. These Nyquist plots were fitted by using CPE model to obtain respective circuit diagram and charge transfer resistance value for Co_3_O_4_/BNS-rGO. The values of fitted parameters for the equivalent circuit diagram are given in Table 1 in which *R*_p_ represents the charge transfer resistance. The Co_3_O_4_ with rGO have catalytic activity but Co_3_O_4_ when coupled with B, N and S doped rGO, it shows higher electrocatalytic activity. This better electrocatalytic activity is due to synergy between Co_3_O_4_ and BNS-rGO.

**Table 1:** Different parameters obtained from analysis of EIS data using CPE model^a^

Material	*R*_s_ (ohms)	*y*_o_ or CPE (μf)	Alpha (*A*)	*W*_d_ (μΩ1FBE)	*R*_P_ (*R*_CT_)(ohms)
Go	773.9	1.31	760	68.83	2.73 × 10^^3^
BNS doped GO	505.9	1.147	675	152.7	2.66 × 10^^3^
Co_3_O_4_	841.8	12.58	591.1	342.8	510.6
Co_3_O_4_/BNS-rGO	40.64	611.7	601.2	686.6	22.24

a Where *R*_s_ = solution resistance, *Y*_o_ = constant phase element, alpha = surface roughness, *W*_d_ = Warburg resistance, *R*_CT_ = charge transfer resistance.

From the comparison of Nyquist plot in Fig. 9c, it is clear that the small semicircle is obtained in case of Co_3_O_4_/BNS rGO as compared to the Co_3_O_4_ and other samples. These results clearly confirm that the composite of cobalt oxide with B, N and S doped rGO has low *R*_CT_ and better conductivity. By combining spinel cobalt oxide with GO which have two electronegative dopants increases the conductivity of electrocatalyst. N and S being more electronegative withdraw electrons from nearby carbon atoms of GO and provide these electrons to metal oxide because the active site in this nanocomposite is transition metal oxide. GO increases the surface area of the catalyst and provides a better site for transportation of electrons. Third dopant boron is less electronegative than carbon attracts oxygen which is the requirement of ORR (availability of oxygen). Boron enhances the adsorption kinetics of oxygen.

## 6. Conclusions

In this study, we synthesize composite of spinel cobalt oxide with B, N and S doped rGO by using hydrothermal method. The electrochemical performance of Co_3_O_4_/BNS-rGO over Co_3_O_4_/rGO has been studied. Due to synergic effect between metal oxide and tri-doped rGO, Co_3_O_4_/BNS-rGO shows better electrocatalytic activity. The synthesized novel catalyst gives maximum current density of −9.97 mA cm^−2^ with better onset potential of 0.99 V *vs.* RHE. These better results of electrocatalyst may be due to electron donation effect of N and S to less electronegative B and presence of graphite oxide sheets as a site for oxygen reduction.

## Author contributions

Afia kanwal: methodology, conceptualization, formal analysis, writing – the original draft, editing. Naila Jabeen: supervision, resources, conceptualization, review, editing. Latif U. Khan: data curation, software, analysis, writing. Amna Bashir: writing, review, editing. Syeda Wishal Bokhari: formal analysis. Zareen Akhter: supervision, resources, project administration.

## Conflicts of interest

The authors affirm that they have no known financial or interpersonal conflicts that would have appeared to have an impact on the research presented in this study.

## Supplementary Material

## References

[cit1] Kiani M., Tian X. Q., Zhang W. (2021). Non-precious metal electrocatalysts design for oxygen reduction reaction in polymer electrolyte membrane fuel cells: recent advances, challenges and future perspectives. Coord. Chem. Rev..

[cit2] Guo Y. G., Hu J. S., Wan L. J. (2008). Nanostructured materials for electrochemical energy conversion and storage devices. Adv. Mater..

[cit3] Jacobson M. Z., Delucchi M. A. (2011). Providing all global energy with wind, water, and solar power, Part I: Technologies, energy resources, quantities and areas of infrastructure, and materials. Energy policy.

[cit4] Sui S., Wang X., Zhou X., Su Y., Riffat S., Liu C. J. (2017). A comprehensive review of Pt electrocatalysts for the oxygen reduction reaction: Nanostructure, activity, mechanism and carbon support in PEM fuel cells. J. Mater. Chem. A.

[cit5] O'hayre R. , Cha S. W., Colella W. and Prinz F. B., Fuel cell fundamentals, John Wiley & Sons, 2016

[cit6] Steele B. C. and Heinzel A., Materials for fuel-cell technologies, in Materials for sustainable energy: a collection of peer-reviewed Research and review articles from nature publishing group, 2011, pp. 224–231, 10.1038/35104620

[cit7] Gómez-Marín A. M., Rizo R., Feliu J. M. (2013). Some reflections on the understanding of the oxygen reduction reaction at Pt (111). Beilstein J. Nanotechnol..

[cit8] Liu M., Zhao Z., Duan X., Huang Y. (2019). Nanoscale structure design for high-performance Pt-based ORR catalysts. Adv. Mater..

[cit9] Wan C., Duan X., Huang Y. (2020). Molecular Design of Single-Atom Catalysts for Oxygen Reduction Reaction. Adv. Energy Mater..

[cit10] Janik M. J., Taylor C. D., Neurock M. (2008). First-principles analysis of the initial electroreduction steps of oxygen over Pt (111). J. Electrochem. Soc..

[cit11] Marković N. M., Schmidt T. J., Stamenković V., Ross P. N. (2001). Oxygen reduction reaction on Pt and Pt bimetallic surfaces: a selective review. Fuel cells.

[cit12] Toda T., Igarashi H., Uchida H., Watanabe M. (1999). Enhancement of the electroreduction of oxygen on Pt alloys with Fe, Ni, and Co. J. Electrochem. Soc..

[cit13] Goswami C., Hazarika K. K., Bharali P. (2018). Transition metal oxide nanocatalysts for oxygen reduction reaction. Mater. Sci. Energy Technol..

[cit14] Chen J., Wu X., Selloni A. (2011). Electronic structure and bonding properties of cobalt oxide in the spinel structure. Phys. Rev. B.

[cit15] Liu J., Bao H., Zhang B., Hua Q., Shang M., Wang J., Jiang L. (2019). Geometric occupancy and oxidation state requirements of cations in cobalt oxides for oxygen reduction reaction. ACS Appl. Mater. Interfaces.

[cit16] Hao Y., Xu Y., Liu J., Sun X. (2017). Nickel–cobalt oxides supported on Co/N decorated graphene as an excellent bifunctional oxygen catalyst. J. Mater. Chem. A.

[cit17] Xie G., Chen B., Jiang Z., Niu X., Cheng S., Zhen Z., Jiang Z. J. (2016). High catalytic activity of Co 3 O 4 nanoparticles encapsulated in a graphene supported carbon matrix for oxygen reduction reaction. RSC Adv..

[cit18] Christy M., Jang H., Nahm K. S. (2015). Cobaltite oxide nanosheets anchored graphene nanocomposite as an efficient oxygen reduction reaction (ORR) catalyst for the application of lithium-air batteries. J. Power Sources.

[cit19] Yang S., Cui G., Pang S., Cao Q., Kolb U., Feng X., Müllen K. (2010). Fabrication of cobalt and cobalt oxide/graphene composites: Towards high-performance anode materials for lithium ion batteries. ChemSusChem.

[cit20] Yuan M., Yang Y., Nan C., Sun G., Li H., Ma S. (2018). Porous Co3O4 nanorods anchored on graphene nanosheets as an effective electrocatalysts for aprotic Li-O2 batteries. Appl. Surf. Sci..

[cit21] Wang Y. J., Fan H., Ignaszak A., Zhang L., Shao S., Wilkinson D. P., Zhang J. (2018). Compositing doped-carbon with metals, non-metals, metal oxides, metal nitrides and other materials to form bifunctional electrocatalysts to enhance metal-air battery oxygen reduction and evolution reactions. Chem. Eng. J..

[cit22] Lu Z., Yang Q., Pan H., Liu Z., Huang X., Chen X., Niu L. (2021). Bifunctional Oxygen Electrocatalysis at Co-B, N, S-Graphene Composite Investigated by Scanning Electrochemical Microscopy at Variable Temperatures and Its Application in Zn-air Battery. Electrochim. Acta.

[cit23] Liang Y., Li Y., Wang H., Zhou J., Wang J., Regier T., Dai H. (2011). Co _3_ O _4_ nanocrystals on graphene as a synergistic catalyst for oxygen reduction reaction. Nat. Mater..

[cit24] Tong Y., Chen P., Zhou T., Xu K., Chu W., Wu C., Xie Y. (2017). A bifunctional hybrid Electrocatalyst for oxygen reduction and evolution: cobalt oxide nanoparticles strongly coupled to B, N-decorated graphene. Angew. Chem., Int. Ed..

[cit25] Ingavale S. B., Patil I. M., Parse H. B., Ramgir N., Kakade B., Swami A. (2018). B, N, S. tri-doped reduced graphite oxide–cobalt oxide composite: a bifunctional electrocatalyst for enhanced oxygen reduction and oxygen evolution reactions. New J. Chem..

[cit26] Liu W., Bao J., Xu L., Guan M., Wang Z., Qiu J., Li H. (2019). NiCo2O4 ultrathin nanosheets with oxygen vacancies as bifunctional electrocatalysts for Zn-air battery. Appl. Surf. Sci..

[cit27] Pu Z., Liu Q., Tang C., Asiri A. M., Qusti A. H., Al-Youbi A. O., Sun X. (2014). Spinel ZnCo2O4/N-doped carbon nanotube composite: A high active oxygen reduction reaction electrocatalyst. J. Power Sources.

[cit28] Liang Y., Li Y., Wang H., Zhou J., Wang J., Regier T., Dai H. (2011). Co 3 O 4 nanocrystals on graphene as a synergistic catalyst for oxygen reduction reaction. Nat. Mater..

[cit29] Buchner F., Eckardt M., Böhler T., Kim J., Gerlach J., Schnaidt J., Behm R. J. (2020). Oxygen Reduction and Evolution on Ni-modified Co3O4 (1 1 1) Cathodes for Zn–Air Batteries: A Combined Surface Science and Electrochemical Model Study. ChemSusChem.

[cit30] Luisetto I., Pepe F., Bemporad E. (2008). Preparation and characterization of nano cobalt oxide. J. Nanopart. Res..

[cit31] Cao P., Wang L., Xu Y., Fu Y., Ma X. (2015). Facile hydrothermal synthesis of mesoporous nickel oxide/reduced graphene oxide composites for high performance electrochemical supercapacitor. Electrochim. Acta.

[cit32] Hummers Jr W. S., Offeman R. E. (1958). Preparation of graphitic oxide. J. Am. Chem. Soc..

[cit33] Xiang C., Li M., Zhi M., Manivannan A., Wu N. (2013). A reduced graphene oxide/Co3O4 composite for supercapacitor electrode. J. Power Sources.

[cit34] Bashir A., Shukla S., Lew J. H., Shukla S., Bruno A., Gupta D., Mhaisalkar S. G. (2018). Spinel Co 3 O 4 nanomaterials for efficient and stable large area carbon-based printed perovskite solar cells. Nanoscale.

[cit35] Lv K., Cheng B., Yu J., Liu G. (2012). Fluorine ions-mediated morphology control of anatase TiO 2 with enhanced photocatalytic activity. Phys. Chem. Chem. Phys..

[cit36] Galande C. , Yalcin S. E., Singh A., Gupta G., Kappera R., Dattelbaum A. M. and Rice University Collaboration, Photo Induced Fluorescence Enhancement and Correlated FTIR of Single Layer Graphene Oxide, APS March Meeting Abstracts, 2014, vol. 2014, p. Y45−007

[cit37] Nandgaonkar A. G., Wang Q., Fu K., Krause W. E., Wei Q., Gorga R., Lucia L. A. (2014). A one-pot biosynthesis of reduced graphene oxide (RGO)/bacterial cellulose (BC) nanocomposites. Green Chem..

[cit38] Jia X., Gao S., Liu T., Li D., Tang P., Feng Y. (2017). Controllable synthesis and bi-functional electrocatalytic performance towards oxygen electrode reactions of Co3O4/N-RGO Composites. Electrochim. Acta.

[cit39] Shahid M. M., Pandikumar A., Golsheikh A. M., Huang N. M., Lim H. N. (2014). Enhanced electrocatalytic performance of cobalt oxide nanocubes incorporating reduced graphene oxide as a modified platinum electrode for methanol oxidation. RSC Adv..

[cit40] Bergmann A., Martinez-Moreno E., Teschner D., Chernev P., Gliech M., De Araújo J. F., Strasser P. (2015). Reversible amorphization and the catalytically active state of crystalline Co_3_O_4_ during oxygen evolution. Nat. Commun..

[cit41] Khan L. U., Jabeen N., Jabbar I., Jamil S., Kanwal A., Akhter Z., Harfouche M. (2021). Investigating local structure of ion-implanted (Ni2+) and thermally annealed rock salt CoO film by EXAFS simulation using evolutionary algorithm. ACS Appl. Energy Mater..

[cit42] Harfouche M., Abdellatief M., Momani Y., Abbadi A., Al Najdawi M., Al Zoubi M., Paolucci G. (2022). Emergence of the first XAFS/XRF beamline in the Middle East: providing studies of elements and their atomic/electronic structure in pluridisciplinary research fields. J. Synchrotron Radiat..

[cit43] Khan L. U., Khan Z. U., Blois L., Tabassam L., Brito H. F., Figueroa S. J. (2023). Strategy to Probe the Local Atomic Structure of Luminescent Rare Earth Complexes by X-ray Absorption Near-Edge Spectroscopy Simulation Using a Machine Learning-Based PyFitIt Approach. Inorg. Chem..

[cit44] Meng L., Liu W., Lu Y., Liang Z., He T., Li J., Yu J. (2023). Lamellar-stacked cobalt-based nanopiles integrated with nitrogen/sulfur co-doped graphene as a bifunctional electrocatalyst for ultralong-term zinc–air batteries. J. Energy Chem..

[cit45] Wei J., Chen H., He J., Huang Z., Qin H., Xiao X., He J. (2023). Cobalt-based N-doped bamboo-like graphene tubes with enhanced durability for efficient oxygen reduction reaction in direct borohydride fuel cell. Carbon.

[cit46] Fajardo S., Ocón P., Rodríguez J. L., Pastor E. (2023). Co supported on N and S dual- doped reduced graphene oxide as highly active oxygen-reduction catalyst for direct ethanol fuel cells. Chem. Eng. J..

